# Analysis of serious adverse events in a pediatric community-acquired pneumonia randomized clinical trial in Malawi

**DOI:** 10.1038/s41598-022-07582-w

**Published:** 2022-03-03

**Authors:** Amy Sarah Ginsburg, Susanne May

**Affiliations:** Clinical Trials Center, University of Washington, Building 29, Suite 250, 6200 NE 74th Street, Seattle, WA 98115 USA

**Keywords:** Health care, Medical research

## Abstract

Amoxicillin is recommended as first-line antibiotic treatment for community-acquired pneumonia, the leading infectious cause of mortality in children aged less than 5 years. We conducted a double-blind, randomized controlled non-inferiority trial comparing 3- to 5-day amoxicillin treatment for non-severe chest-indrawing pneumonia in HIV-negative children aged 2 to 59 months in Malawi. In a secondary analysis, we assessed the frequency of serious adverse events (SAEs) during the trial to evaluate the safety of treatment with amoxicillin. Enrolled children with non-severe chest-indrawing pneumonia were randomized to either 3- or 5-day amoxicillin and followed for 14 days to track clinical outcomes. In addition to evaluation for treatment failure (primary endpoint, day 6), relapse, and study drug adherence, children were assessed for adverse events, including SAEs, which were managed per local standard clinical practice until resolution or stabilization. Between March 2016 and April 2019, 3000 children were enrolled, with male and younger children (aged less than 24 months) demonstrating more SAEs (10.3% for males vs 8.1% for females, *p* = 0.04; 10.0% for 2–6 months, 10.8% for 7–11 months, 9.7% for 12–23 months and 5.6% for 24–59 months, *p* = 0.01). The most common SAEs were progression of or recurrent pneumonia (220 SAEs in 217 children), acute gastroenteritis (14 SAEs in 14 children), and fever (8 SAEs in 8 children); however, there were no significant or substantive differences in the percentage of children with pneumonia-related, acute gastroenteritis, or fever SAEs noted between the 3- versus 5-day amoxicillin treatment groups. In our pediatric community-acquired pneumonia trial evaluating amoxicillin treatment, there were relatively few SAEs overall and very few attributed to amoxicillin. Duration of amoxicillin treatment did not impact the frequency of SAEs. We found male and younger children appear to be more vulnerable to SAEs in our trial; however, our data support previous data demonstrating the safety of amoxicillin use in children with pneumonia.

Clinical trial registration: ClinicalTrials.gov (NCT02678195).

## Introduction

Acute respiratory tract infections are the most common reason for patient encounters in pediatric care and for antibiotic prescriptions^1,2^. Of all medication classes prescribed to children in both hospital and community settings, antibiotics are responsible for the majority of adverse drug events^3^. In addition to being one of the most commonly prescribed antibiotics globally, amoxicillin accounts for among the most frequent adverse drug events seen in emergency departments^1,2,4^. Amoxicillin is recommended as first-line antibiotic treatment of community-acquired pneumonia, the leading infectious cause of mortality in children less than five years of age^5^. Generally well-tolerated and safe, amoxicillin has important side effects which include diarrhea, nausea, vomiting, fever, and rash as well as rarer but more serious side effects such as abnormal liver function tests, interstitial nephritis, seizures, and Stevens-Johnson syndrome, all of which could lead to serious adverse events (SAEs)^6–9^. To evaluate the safety of treatment with amoxicillin in children enrolled in a pediatric community-acquired pneumonia treatment trial, we conducted a secondary analysis and assessed the frequency of SAEs.

## Methods

In a prospective double-blind randomized controlled non-inferiority clinical trial carried out in a malaria-endemic region of Malawi, we enrolled HIV-uninfected children aged 2 to 59 months with non-severe chest-indrawing pneumonia and compared treatment with 5-day versus 3-day oral amoxicillin dispersible tablets (Appendix 1. Study protocol)^10^. Following initial study drug administration, children were typically observed in-hospital for 2 days, discharged on day 3 if no treatment failure criteria (World Health Organization general danger sign (lethargy or unconsciousness, convulsions, vomiting everything, inability to drink or breastfeed), sign of severe respiratory distress (grunting, nasal flaring, head nodding, or chest indrawing), or hypoxemia) were present, and followed for 14 days to track clinical outcomes. At all scheduled (days 2, 4, 6, and 14), and unscheduled follow-up visits, children were assessed for treatment failure (primary endpoint, day 6 with a relative non-inferiority margin of 1.5 times the treatment failure rate in the 5-day amoxicillin group) or relapse, and study drug adherence^11^. All adverse events were assessed and managed per local standard clinical practice, documented, and followed and treated until resolution or stabilization. All SAEs were reported to the study safety team for review within 24 h and a detailed SAEs report was completed. The United States National Institutes of Health Division of AIDS adverse event grading system was used to assess severity of the event^12^. For continuous baseline characteristics, averages between children reporting any SAEs versus children reporting no SAEs were compared using t-tests. Differences in the number of children who reported SAEs for different baseline characteristics as well as safety outcomes were assessed using chi-square statistics or, if the expected cell count was less than five, Fisher’s exact tests. No adjustments were made for multiple comparisons and complete case analyses were used. A data safety monitoring board was established to routinely and independently assess child safety throughout the trial. The trial was approved by Western Institutional Review Board; College of Medicine Research and Ethics Committee; and Malawi Pharmacy, Medicines and Poisons Board; and was registered with ClinicalTrials.gov (NCT02678195; 09/02/2016).

### Ethical approval and consent to participate

The study was conducted in accordance with the International Conference on Harmonisation, Good Clinical Practice and the Declaration of Helsinki 2008, and was approved by the Western Institutional Review Board in the state of Washington, USA; the College of Medicine Research and Ethics Committee, Blantyre, Malawi; and the Malawi Pharmacy, Medicines and Poisons Board. Written informed consent for study participation was obtained from the caregiver or legal guardian of each study participant.

## Results

Conducted between March 2016 and April 2019 at Kamuzu Central Hospital and Bwaila District Hospital in Lilongwe, Malawi, 3000 children were enrolled and included in this analysis. As reported previously, children receiving 3 days of amoxicillin had a 5.9% (85/1442 with outcome data) treatment failure rate by Day 6, within the non-inferiority margin of those receiving 5 days of amoxicillin (5.2% (75/1456) treatment failure rate), with an adjusted absolute difference of 0.75% and 95% confidence interval (CI) − 0.92%, 2.41%^10^. In addition to presenting with more severe pneumonia, male and younger children (aged less than 24 months) demonstrated more SAEs (10.3% for males vs 8.1% for females, *p* = 0.04; 10.0% for 2–6 months, 10.8% for 7–11 months, 9.7% for 12–23 months and 5.6% for 24–59 months, *p* = 0.01) (Table 1). The most common SAEs were progression or recurrent pneumonia (220 SAEs in 217 children), acute gastroenteritis (14 SAEs in 14 children), and fever (8 SAEs in 8 children) (Table 2); however, there were no significant or substantive differences in the percentage of children with pneumonia-related, acute gastroenteritis, or fever SAEs noted between the 5- versus 3-day amoxicillin treatment groups. Furthermore, when comparing the grade of SAE severity between the amoxicillin treatment groups, there were no significant or substantive differences noted (Table 3). Six children had life-threatening SAEs (all with danger sign pneumonia on days 1, 2 or 3) and three children died (one with pneumonia on day 5, one with danger sign pneumonia on day 5, and one with gastroenteritis on day 11). Nine children had two SAEs and no child had more than two SAEs. Only two SAEs were determined to be possibly (one with danger sign pneumonia) or probably (one with fever) related to amoxicillin.

**Table 1:** Number of children for whom serious adverse events (SAEs) were reported by baseline characteristics.

	Number with SAEs or average by SAE status		*p* value
Overall	279/3000	9.3%	
**Sex**			
Males	170/1653	10.3%	0.04
Females	109/1347	8.1%	
**Age (years)**			0.01
2–6	116/1155	10.0%	
7–11	63/581	10.8%	
12–23	69/713	9.7%	
24–59	31/551	5.6%	
**Respiratory rate (breaths/min)**			0.37
< 40	28/406	6.9%	
40–49	100/1034	9.7%	
50–59	100/1090	9.2%	
60–69	45/416	10.8%	
≥ 70	6/54	11.1%	
**Oxygen saturation (%)**			0.38
< 90	0	0	
90–92	2/10	16.7%	
≥ 93	277/2988	9.3%	
**Axillary temperature (°C)**			0.49
< 38	198/2074	9.6%	
≥ 38	81/926	8.8%	
**Heart rate (beats/min)**	Mean (SD)		0.31
Without SAEs (2721)	150.2 (16.3)		
With SAEs (279)	151.3 (14.9)		
**Weight-for-height z-score**			0.95
Without SAEs (2721)	0.83 (1.3)		
With SAEs (279)	0.82 (1.3)		
**Mid-upper arm circumference (cm)**			0.11
≤ 12.5	18/136	13.2%	
> 12.5	261/2864	9.1%	
**Malaria (rapid diagnostic test)**			0.38
Yes	21/269	7.8%	
No	258/2731	9.5%	
**Diarrhea**			0.77
Yes	40/413	9.7%	
No	239/2587	9.2%

**Table 2:** Number of children with serious adverse events (SAEs) by treatment group.

	3 days amoxicillin	5 days amoxicillin	*p* value
**Fast-breathing pneumonia**			0.58
No	1480	1489	
Yes	17	14	
**Chest-indrawing pneumonia**			0.27
No	1437	1454	
Yes	60	49	
**Danger sign pneumonia**			0.86
No	1448	1452	
Yes	49	51	
**Chest x-ray confirmed pneumonia**			0.20
No	1490	1500	
Yes	7	3	
**Acute gastroenteritis**			0.59
No	1489	1497	
Yes	8	6	
**Fever**			0.73*
No	1494	1498	
Yes	3	5	

*Based on Fisher’s exact test.

**Table 3:** Characteristics of serious adverse events (SAEs) by treatment group.

	3 days amoxicillin	5 days amoxicillin	Total
Highest grade (per child) (*p* = 0.20)*	147	132	279
Mild—1	0	3	3
Moderate—2	20	17	37
Severe—3	121	108	229
Life-threatening—4	5	1	6
Death—5	1	2	3
Missing	0	1	1

Relatedness of SAE**	3 days amoxicillin	5 days amoxicillin	
Not related	146	129	
Probably not related	1	0	
Possibly related	0	1	
Probably related	0	1	
Missing	0	1	

*Based on Fisher’s exact test.

**No formal testing done because of small numbers.

## Discussion

In our pediatric community-acquired pneumonia trial evaluating amoxicillin treatment, there were relatively few SAEs overall and very few attributed to amoxicillin. In addition to finding 3 days of amoxicillin treatment for chest-indrawing pneumonia non-inferior to 5 days among HIV-uninfected Malawian children, the duration of amoxicillin treatment did not appear to impact the frequency of SAEs. The low rate of SAEs may be partly a consequence of the high level of supportive care and monitoring the enrolled children received and/or of the study eligibility criteria that excluded HIV-seropositive children and those with signs of severe disease or acute malnutrition. However, since common harms from antibiotics are poorly quantified and frequently not reported, reviewing SAEs frequency is important to assess potential serious adverse impacts of amoxicillin used for treatment of non-severe chest-indrawing pediatric pneumonia.

In our trial, we found male and younger children appear to be more vulnerable to SAEs. Male sex and younger age have been shown to be associated with more severe disease presentations and increased mortality^13,14^. Furthermore, amoxicillin adverse events have shown to be higher for younger children^4^. Contributing factors may include increased susceptibility to antibiotic allergy among younger children and caregivers having a lower threshold for seeking care for young children they perceive to be more vulnerable^15^.

Limitations to our study included the small sample size of children with SAEs and the limited duration of follow-up (2 weeks). Potential bias may have been introduced with inaccurate self-reporting of adherence which, if present, could have resulted in making the groups look more similar for primary, secondary, and safety outcomes. In addition, the study did not obtain laboratory or imaging confirmation regarding the diagnosis of chest-indrawing pneumonia. As a result, we may have observed fewer SAEs than would have been the case otherwise.

While our clinical trial data further support previous data demonstrating the safety of amoxicillin use in children with pneumonia^16–18^, we believe it is important to ensure adequate and complete safety monitoring and to report and disseminate the findings, particularly from studies involving children. The dissemination of these data as well as active and passive surveillance data that help quantify and qualify the benefits versus risks of antibiotic treatment in young children is critical to better understanding the optimal use cases in young children.
